# Correlation between Clinical and Wastewater SARS-CoV-2 Genomic Surveillance, Oregon, USA

**DOI:** 10.3201/eid2809.220938

**Published:** 2022-09

**Authors:** Devrim Kaya, Rebecca Falender, Tyler Radniecki, Matthew Geniza, Paul Cieslak, Christine Kelly, Noah Lininger, Melissa Sutton

**Affiliations:** Oregon State University, Corvallis, Oregon, USA (D. Kaya, T. Radniecki, M. Geniza, C. Kelly);; Oregon Health Authority, Portland, Oregon, USA (R. Falender, P. Cieslak, N. Lininger, M. Sutton)

**Keywords:** COVID-19, respiratory infections, severe acute respiratory syndrome coronavirus 2, SARS-CoV-2, SARS, coronavirus disease, zoonoses, viruses, coronavirus, wastewater-based epidemiology, wastewater surveillance, genomic surveillance, Oregon, United States

## Abstract

SARS-CoV-2 variant proportions in a population can be estimated through genomic sequencing of clinical specimens or wastewater samples. We demonstrate strong pairwise correlation between statewide variant estimates in Oregon, USA, derived from both methods (correlation coefficient 0.97). Our results provide crucial evidence of the effectiveness of community-level genomic surveillance.

Genomic surveillance to detect SARS-CoV-2 variants has become a critical component of monitoring the virus over time. Both patient- and community-level surveillance through the sequencing of clinical specimens and wastewater samples can detect variants and estimate their proportions in a population. Sequencing wastewater for SARS-CoV-2 variants is an emerging science that offers several advantages over patient-level surveillance, including reduced cost and tracking of cases regardless of symptoms or testing access (*1*,*2*), but few data have demonstrated comparable effectiveness in estimating variant proportions over time (*3*–*5*). We describe the correlation between SARS-CoV-2 variant proportions detected through sequencing of wastewater samples and clinical specimens in Oregon, USA, during February 7, 2021–February 26, 2022.

In brief, 24-hour composite samples were collected >1 time each week from wastewater treatment facility influents for sequencing. We quantified SARS-CoV-2 RNA concentrations via droplet digital reverse transcription PCR and sequenced positive samples on a HiSeq 3000 or NextSeq 2000 sequencer (Illumina, https://www.illumina.com) by using the Swift Amplicon SARS-CoV-2 Panel and Swift Amplicon Combinatorial Dual indexed adapters (Integrated DNA Technologies [IDT] Swift Biosciences, https://www.idtdna.com), according to the manufacturers’ protocols, as previously described (*6*).

During each surveillance week of the study period, we used clinical specimen and wastewater sample data to estimate the proportion of SARS-CoV-2 variants according to US Centers for Disease Control and Prevention variant of concern (VOC) designations (*7*). We defined the circulation period of each variant by its earliest and latest detections in either wastewater or clinical specimens; we included estimated proportions of 0 that fell within a variant’s circulation period in all analyses. To estimate variant proportions using clinical data, we divided the number of specimens for each variant by the total number of SARS-CoV-2–positive specimens from Oregon submitted to the GISAID database (https://www.gisaid.org) by surveillance week (*8*). To estimate variant proportions using wastewater data, we divided the statewide gene copies of each variant by the total gene copies of all variants by surveillance week. To derive the denominator, we normalized the SARS-CoV-2 concentration to wastewater influent flow at each facility and summed the values for all facilities by surveillance week. To derive the numerator, we multiplied the normalized SARS-CoV-2 concentration by the proportion of sequence reads for each SARS-CoV-2 variant detected at each facility and summed the values for all facilities by surveillance week.

We used the Pearson correlation coefficient (*r*) to assess the relationship between the statewide weekly estimated proportions of each VOC detected in clinical specimens and wastewater samples. We used simple linear regression with a least-squares regression line to assess goodness of fit (R^2^) and considered p<0.05 statistically significant. We used Stata version 17.0 (StataCorp LLC, https://www.stata.com) for all analyses.

Of 488,308 confirmed COVID-19 cases in Oregon during the study period, 38,386 (7.9%) clinical samples were sequenced and submitted to the GISAID database. Of 2,948 wastewater samples collected from 42 communities, 2,852 (97%) tested positive for SARS-CoV-2 and 2,749 (96%) were sequenced. We included 233 pairs of estimated proportions in the correlation analysis and rounded all estimates to 0.001. 

Overall, statewide weekly estimated percentages of each SARS-CoV-2 variant detected in clinical specimens were strongly associated with those from wastewater samples; *r* was 0.97 for all variants (p<0.0001) (Figure). However, *r* fluctuated by SARS-CoV-2 variant, from 0.61 for Beta to 0.98 for Delta, and we noted a general increasing trend in *r* as total variant proportions increased (Table). A scatter plot demonstrated a linear relationship between estimated percentages of each variant derived from clinical specimens and wastewater samples (Figure, panel B). The conditional SD was greatest for proportion estimates of 0.2–0.6. Simple linear regression demonstrated a strong linear relationship between estimated proportions derived from both genomic surveillance data sources (R^2^ = 0.94; p<0.0001).

**Figure:**
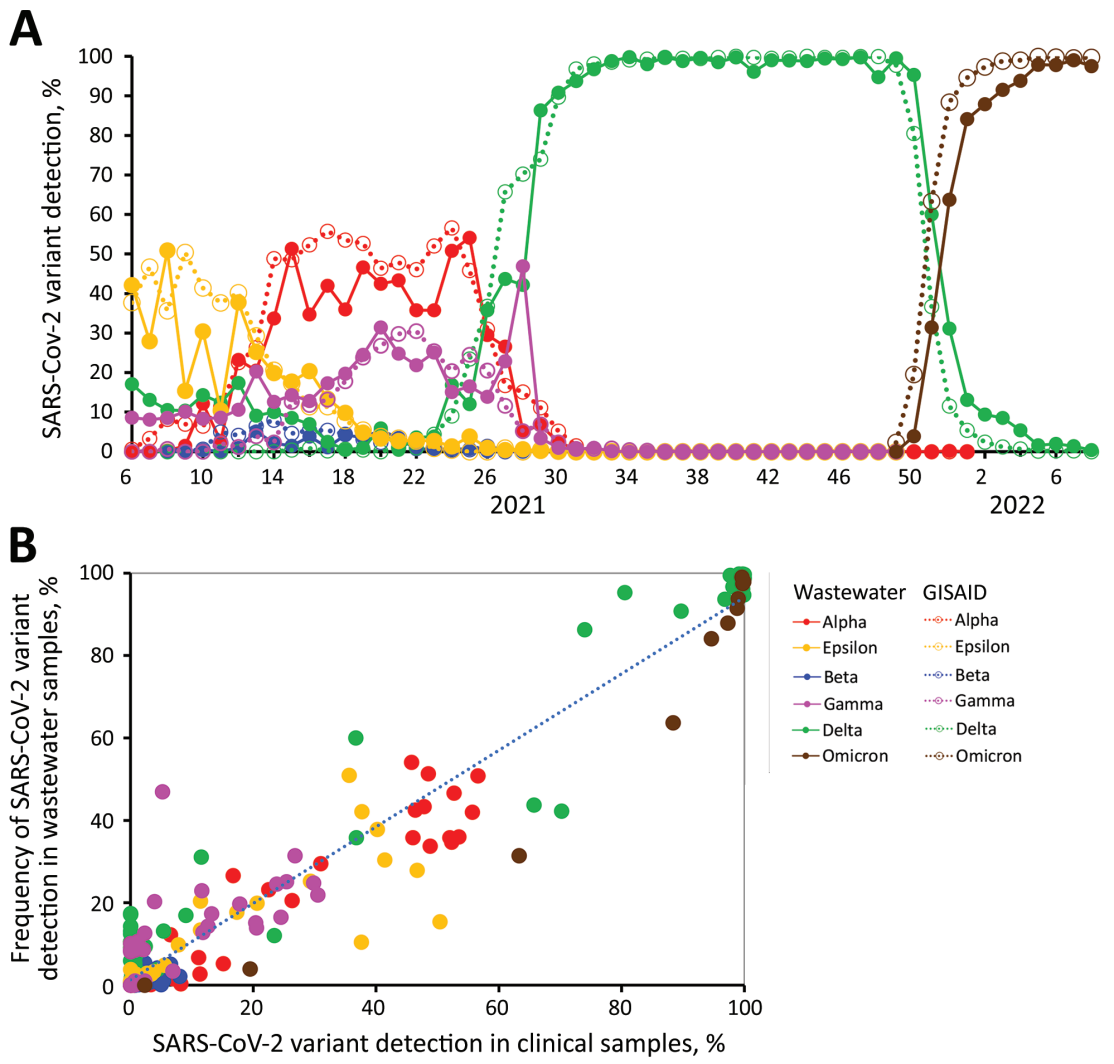
Comparison of SARS-CoV-2 genomic sequence data from confirmed COVID-19 case clinical specimens and wastewater samples collected in Oregon, USA, February 6, 2021–February 26, 2022. A) Percentages of different SARS-CoV-2 variants detected during each epidemiologic week. B) Scatter plot comparing variant detection frequency by sample type. Clinical specimens were retrieved from the GISAID database (https://www.gisaid.org).

**Table:** Correlation between estimated SARS-CoV-2 variant proportions detected in clinical specimens and wastewater samples, Oregon, USA, February 7, 2021–February 26, 2022*

Variant	*r*	R^2^	p value	No. (%) pairwise observations included in correlation
All	0.97	0.94	<0.0001	233 (100)
Alpha B.1.1.7†	0.96	0.93	<0.0001	48 (20.6)
Beta B.1.351	0.61	0.38	0.0003	30 (12.9)
Delta B.1.617.2‡	0.98	0.97	<0.0001	55 (23.6)
Epsilon B.1.427/429	0.86	0.74	<0.0001	44 (18.9)
Gamma P.1§	0.71	0.50	<0.0001	44 (18.9)
Omicron B.1.1.529¶	0.97	0.93	<0.0001	12 (5.2)

*Sequence data for clinical samples were retrieved from GISAID (https://www.gisaid.org). *r*, Pearson correlation coefficient; R^2^, simple linear regression with a least-squares regression line to assess model fit. †B.1.1.7 includes all Q sublineages.  ‡B.1.617.2 includes all AY sublineages. §P.1 includes all P.1 sublineages. ¶B.1.1.529 includes all BA sublineages.

Our pairwise correlation analysis demonstrates the effectiveness of wastewater sequencing for estimating SARS-CoV-2 variant proportions at the statewide level over time and at varying prevalences. Overall, the association between estimates of variant proportions produced from clinical specimens and wastewater samples was strong. However, correlations varied by VOC and were weakest for the least prevalent variants. 

A limitation of wastewater surveillance is that it excludes populations without access to municipal sewer service (i.e., those with septic systems); therefore, it might not be generalizable to all populations within a state. However, for other areas, leveraging wastewater surveillance for SARS-CoV-2 genomic surveillance offers several advantages over estimating variant proportions from clinical specimens. Because wastewater surveillance does not rely on healthcare access, testing acceptance, and molecular testing availability, it likely provides more robust and less biased estimates than sequencing of clinical specimens. Thus, wastewater genomic surveillance could prove valuable in surveillance for many other pathogens of public health concern.

## References

[1] Hart OE, Halden RU. Computational analysis of SARS-CoV-2/COVID-19 surveillance by wastewater-based epidemiology locally and globally: Feasibility, economy, opportunities and challenges. Sci Total Environ. 2020;730:138875. 10.1016/j.scitotenv.2020.138875 32371231 PMC7175865

[2] Daughton CG. Wastewater surveillance for population-wide Covid-19: The present and future. Sci Total Environ. 2020;736:139631. 10.1016/j.scitotenv.2020.139631 32474280 PMC7245244

[3] Crits-Christoph A, Kantor RS, Olm MR, Whitney ON, Al-Shayeb B, Lou YC, et al. Genome sequencing of sewage detects regionally prevalent SARS-CoV-2 variants. MBio. 2021;12:e02703–20. 10.1128/mBio.02703-20 33468686 PMC7845645

[4] Agrawal S, Orschler L, Schubert S, Zachmann K, Heijnen L, Tavazzi S, et al. Prevalence and circulation patterns of SARS-CoV-2 variants in European sewage mirror clinical data of 54 European cities. Water Res. 2022;214:118162. 10.1016/j.watres.2022.118162 35193077 PMC8817224

[5] Rios G, Lacoux C, Leclercq V, Diamant A, Lebrigand K, Lazuka A, et al. Monitoring SARS-CoV-2 variants alterations in Nice neighborhoods by wastewater nanopore sequencing. Lancet Reg Health Eur. 2021;10:100202. 10.1016/j.lanepe.2021.100202 34423327 PMC8372489

[6] Sutton M, Radniecki TS, Kaya D, Alegre D, Geniza M, Girard A-M, et al. Detection of SARS-CoV-2 B.1.351 (Beta) variant through wastewater surveillance before case detection in a community, Oregon, USA. Emerg Infect Dis. 2022;28:1101–9. 10.3201/eid2806.211821 35452383 PMC9155900

[7] Centers for Disease Control and Prevention. SARS-CoV-2 variant classifications and definitions 2022 Apr 26 [cited 2022 Apr 29]. https://www.cdc.gov/coronavirus/2019-ncov/variants/variant-classifications.html

[8] Elbe S, Buckland-Merrett G. Data, disease and diplomacy: GISAID’s innovative contribution to global health. Glob Chall. 2017;1:33–46. 10.1002/gch2.1018 31565258 PMC6607375

